# Schimke immunoosseous dysplasia (SIOD): Delayed onset of rare disease and novel variant

**DOI:** 10.5339/qmj.2025.121

**Published:** 2025-12-15

**Authors:** Mostafa Elshirbeny, Awais Nauman, Essa Abuhelaiqa, Hassan Almalki

**Affiliations:** 1 Nephrology Division, Medicine Department, Hamad General Hospital, Hamad Medical Corporation, Doha, Qatar *Email: melshirbeny@hamad.qa

**Keywords:** Schimke immunoosseous dysplasia, proteinuria, end-stage kidney disease, Qatar

## Abstract

**Background:**

Schimke immunoosseous dysplasia (SIOD) is a condition marked by spondyloepiphyseal dysplasia (SED), leading to short stature, nephropathy, and T-cell immunodeficiency.

**Case presentation:** A 15-year-old male was referred to the nephrology clinic with a gradual onset of lower-limb swelling. Clinical examination revealed short stature. Laboratory studies revealed renal impairment and nephrotic-range proteinuria. Kidney biopsy showed global sclerosis in 3 of 28 glomeruli, segmental sclerosis in 16 of 28 glomeruli, and 90% foot-process effacement on electron microscopy. A skeletal survey showed flattened thoracolumbar vertebral bodies, a characteristic feature of SIOD. Flow cytometry revealed a low CD4 count. Whole-exome sequencing confirmed that the proband was homozygous for the p.(R611C) variant in the *SMARCAL1* gene. The patient was treated with a calcineurin inhibitor (CNI), angiotensin receptor blockers (ARBs), and prophylactic anticoagulation. Initially, he experienced improvement in serum albumin levels and proteinuria. However, his urine protein and creatinine levels subsequently increased, prompting discontinuation of CNI. The patient progressed to end-stage kidney disease (ESKD) and required hemodialysis 18 months after the initial presentation.

**Discussion:** This report describes a case of SIOD, a rare multisystem disorder characterized by short stature due to SED and nephrotic syndrome, distinguishing it from other hereditary nephrotic syndromes. The patient presented unusually in adolescence with nephrotic-range proteinuria and skeletal abnormalities, representing a rarely reported juvenile variant. Genetic testing revealed a novel homozygous *SMARCAL1* mutation, p.(R611C), which was confirmed in his younger sibling. However, the phenotypic expression differed, reflecting the weak genotype–phenotype correlation in SIOD. Unlike many SIOD cases, our patient did not experience recurrent infections despite abnormal immune parameters, suggesting a milder immunodeficiency and making kidney transplantation with cautious immunosuppression a feasible option. Current therapeutic challenges include the lack of effective disease-specific treatments, limited success with conventional transplantation due to infection and malignancy risks, and emerging but logistically complex approaches such as combined stem-cell and kidney transplantation.

**Conclusion:**

SIOD is a rare genetic disorder with variable presentation and outcomes. Our patient, carrying a novel *SMARCAL1* mutation and presenting with juvenile-onset disease, progressed to ESKD but had only mild immune dysfunction, making kidney transplantation a potential treatment option. Early recognition is essential to avoid unnecessary treatments, guide supportive care, and enable timely referral for advanced therapies. Clinicians should consider SIOD in patients presenting with short stature and nephrotic syndrome to optimize outcomes.

## 1. INTRODUCTION

Schimke immunoosseous dysplasia (SIOD) is an ultra-rare disorder, with an estimated incidence of 1 in 1–3 million live births.^1^ It is a multisystem hereditary condition characterized by skeletal abnormalities, nephrotic syndrome,^2^ and immune dysfunction. SIOD is inherited in an autosomal recessive manner and results from biallelic mutations in the *SMARCAL1* gene.^3^ Diagnosis is typically based on clinical suspicion in children presenting with steroid-resistant nephrotic syndrome and short stature. Progression to end-stage kidney disease (ESKD) is common. T-cell dysfunction in SIOD poses significant challenges for kidney transplantation, primarily due to an increased risk of infections and malignancies. Currently, there is no definitive treatment for the renal complications of SIOD, apart from dialysis in cases of ESKD.^4^ However, recent reports suggest that kidney transplantation may be feasible with reduced-dose immunosuppression or through sequential bone marrow and kidney transplantation.^5,6^

Consanguineous marriages are highly prevalent in the Middle East, including the Gulf countries, with estimated rates ranging from 20% to 50%, and first-cousin unions being the most common form.^7^ This high prevalence increases the probability of autosomal recessive disorders, including SIOD. We report a case of SIOD referred to the nephrology clinic at Hamad General Hospital, the largest public tertiary care facility in Qatar, with informed consent obtained from the patient. The publication of this manuscript was approved by the Medical Research Center at Hamad Medical Corporation (MRC-04-23-769).

## 2. CASE PRESENTATION

A 15-year-old male, the second child of healthy consanguineous Qatari parents, who had been under follow-up at a primary health care center in Qatar, was referred to the nephrology clinic at Hamad General Hospital with a three-month history of gradually progressive lower-limb swelling and facial puffiness. The patient was able to keep up with age-appropriate academics and maintain normal levels of physical activity. Clinical examination revealed short stature (height: 145 cm), mild hypertension, and a soft ejection systolic murmur. Laboratory results revealed the following: white blood cells 2.8×10^3^/μL, lymphocytes 75/ml (lymphopenia), hemoglobin 11.8 g/dL, serum creatinine 115 μmol/L, 24-hour urine protein 4.94 g, thyroid-stimulating hormone (TSH) 11.00 mIU/L, and free thyroxin 4 (FT4) 10.5 pmol/L. Serologies for antinuclear antibody (ANA), antineutrophil cytoplasmic antibody (ANCA), hepatitis B virus (HBV), and hepatitis C virus (HCV) were negative. Minimal change disease is the most common cause of childhood nephrotic syndrome; therefore, a trial of steroids is recommended without the need for a kidney biopsy.^8^ However, in this case, the presence of short stature, gradual onset of symptoms, and lymphopenia prompted us to consider SIOD as the unifying diagnosis for the clinical presentation. A kidney biopsy was performed in the outpatient setting at four weeks, followed by a skeletal survey at five weeks, and lymphocyte flow cytometry at eight weeks after presentation to the nephrology clinic, to assess for abnormalities associated with SIOD.

**Figure 1:**
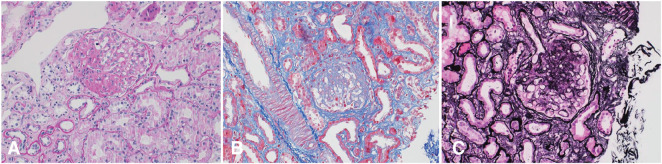
Light microscopy of the patient’s kidney biopsy. (A) A glomerulus with segmental sclerosis (PAS stain, ×200). (B) A glomerulus with segmental sclerosis and mild interstitial fibrosis (Masson Trichrome stain, ×200). (C) A glomerulus with segmental sclerosis as highlighted by silver stain (Jones-Silver stain, ×200).

Kidney biopsy revealed that 3 of 28 glomeruli were globally sclerotic, while 16 of 28 glomeruli showed segmental sclerosis with tuft adhesion to Bowman’s capsule (Figures 1A–C). Electron microscopy (EM) revealed two segmentally sclerosed glomeruli, no deposits, normal mesangial regions, intact basement membrane, and approximately 90% foot-process effacement.

**Figure 2:**
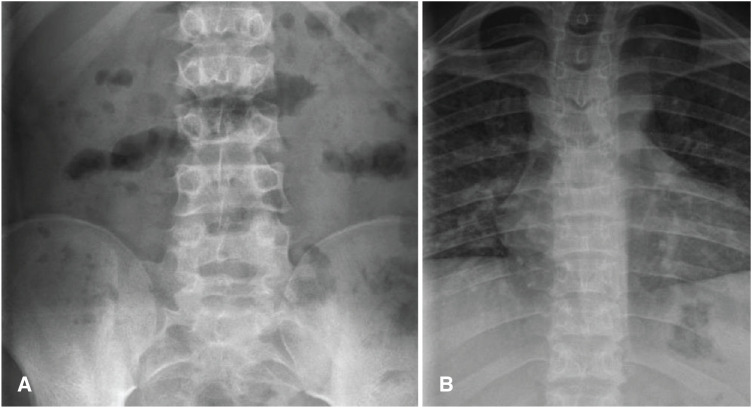
Bone X-ray findings in a patient with Schimke Immunoosseous dysplasia. (A) Lumbar spine and (B) Thoracis spine showing relatively flattened vertebral bodies with irregularly outlined endplates.

Skeletal survey showed flattened thoracolumbar vertebral bodies consistent with SIOD (Figures 2A and B); however, X-rays of the skull, femur, and hips reveal no abnormalities. Echocardiography performed for evaluation of the murmur was unremarkable except for a suspected patent foramen ovale (PFO). Immunology flow cytometry revealed a low CD4 count of 17 (normal: 25–48) and a CD4/CD8 ratio of 0.59. The first-line treatment for childhood nephrotic syndrome is glucocorticoids; however, the phenotypic features of SIOD indicated a genetic cause, and steroids were therefore not administered. Following the availability of the biopsy results, anti-proteinuric therapy with an angiotensin receptor blocker (ARB) and a calcineurin inhibitor (CNI), specifically tacrolimus, was initiated, and genetic testing was requested. The results confirmed that the patient and his sister are homozygous for the p.(R611C) variant in the *SMARCAL1* gene, while the proband’s father, mother, and one sibling are heterozygous carriers. Two other siblings do not harbor the p.(R611C) (c.1831 C>T) variant in the SMARCAL1 gene.

After two months of treatment with ARBs and CNIs, the patient showed some improvement in serum albumin and proteinuria; however, the 24-hour urine protein and serum creatinine later began to increase consistently. To minimize the nephrotoxic effects of CNIs, the tacrolimus dose was reduced, and the patient was maintained on ARBs, a sodium–glucose cotransporter-2 inhibitor (SGLT2i), warfarin, and levothyroxine. Serum creatinine continued to rise during follow-up, and the patient progressed to ESKD, requiring initiation of hemodialysis 18 months after initial presentation. The patient’s younger sister, who was also found to be homozygous for p.(R611C), has short stature and proteinuria, with a urine PCR of 30 mg/mmol, although markedly lower than that of the patient.

## 3. DISCUSSION

Here, we present a case of SIOD, diagnosed based on the classical features of short stature and nephrotic syndrome.^9^ Nephrotic syndrome is the most common feature that brings these young patients to medical attention.^10^ Short stature, resulting from spondyloepiphyseal dysplasia (SED), serves as an important clinical clue suggestive of SIOD. Although other hereditary nephrotic syndromes—such as Galloway–Mowat syndrome,^11^ coenzyme Q10 deficiency syndromes,^12^ and Pearson syndrome^13^—can also present with short stature. None exhibits the characteristic skeletal features of SED.^14^ In addition, short stature has been reported in more than 90% of cases at the time of diagnosis^15^ and usually precedes renal dysfunction by 1–5 years.^16^

Our case exhibits unusual findings compared with the previously reported SIOD cases. Firstly, presentation during the teenage years is rare and has been reported in only a few cases.^17,18^ This is termed the juvenile variant, in contrast to the childhood or infantile forms, which are often more severe.^4^ A comparison of our case with other SIOD cases with delayed presentation is shown in Table 1. Secondly, the p.(R611C) (c.1831 C>T) variant in the SMARCAL1 gene is a novel variant and has not been previously reported in SIOD. And thirdly, despite abnormal CD4 counts and CD4:CD8 ratios, our patient did not experience recurrent infections, which have been reported in 60–80% of the previously documented cases.^19^ This has important implications as impaired immunity significantly increases the risk of infections and malignancy post-transplant, often rendering these patients poor transplant candidates. However, in individuals with mild immune dysfunction, as in our case, kidney transplantation with low-dose immunosuppression remains a viable treatment option for ESKD.^5^

SIOD is caused by mutations in the *SMARCAL1* gene, which encodes the SWI/SNF-related, matrix-associated, actin-dependent regulator of chromatin subfamily A-like protein-1 (*SMARCAL1*)—an annealing helicase that unwinds and re-anneals DNA strands using ATP hydrolysis as an energy source.^20^ Functional analyses of SIOD-associated mutations have shown that most of them impair the adenosine triphosphatase (ATPase) activity of the *SMARCAL1* protein. The R611C variant in exon 11 of the *SMARCAL1* gene affects ATP hydrolysis and is therefore expected to impair protein function.^4^ However, to the best of our knowledge, this variant has not been previously described in the literature. Reference laboratory in silico analyses suggest that this missense mutation has a deleterious effect on protein function, although we do not have access to the specific in silico methods used by the laboratory. Pathogenicity is further supported by the presence of clinical features—namely, proteinuria and short stature—in the younger sibling of our patient, who harbors the same homozygous mutation.

The correlation between SIOD genotype and phenotype is weak, possibly due to variability in gene expression, oligogenic inheritance, or environmental factors.^21^ A report from Italy described three siblings with markedly different disease courses: one brother was diagnosed at 8 years of age and died at 12 due to complications of severe renal impairment, another brother developed ESKD at 22 years of age, and their sister maintained normal kidney function throughout the follow-up period.^22^ In a more recent report of two sisters, the genetic study revealed the same homozygous *SMARCAL1* gene mutation in both; yet their renal phenotypes differed, with one sister developing ESKD within four months while the other maintained normal renal function.^23^ This may explain why the younger sister of our patient, despite carrying the same genetic variant, has only minimal proteinuria at the age of 10 and maintains normal kidney function.

Currently, no effective treatment for SIOD exists. Early diagnosis is important to avoid unnecessary immunosuppressive treatment, which exposes patients to a high risk of severe infections. Dialysis is often initiated due to rapid deterioration of renal function, but long-term dialysis carries a significant risk of atherosclerosis and can accelerate the progression of cardiovascular complications.^9^ Kidney transplantation outcomes in patients with SIOD have been disappointing due to the heightened risk of infections and malignancies associated with chronic viral infections.^24^ Nonetheless, a ray of hope for these patients has emerged with the development of a sequential stem-cell—kidney transplantation program at Stanford Children’s Health, in which a haploidentical donor is first used for stem-cell transplantation, followed by kidney transplantation a few months later. This approach obviates the need for immunosuppression.^6^ We had referred our patient to Stanford Children’s Health; however, unfortunately, this option could not be pursued due to the absence of a suitable haploidentical donor.

## 4. CONCLUSION

We report an unusual presentation of a patient with SIOD carrying a novel *SMARCAL1* gene variant, which, to our knowledge, has not been previously described in medical literature. Unlike the classical form of SIOD, which typically presents early-onset nephrotic syndrome, severe infections, and profound skeletal abnormalities, our patient exhibited a milder phenotype characterized by delayed onset of renal symptoms and the absence of recurrent or severe infections. This atypical clinical course broadens the phenotypic spectrum of SIOD and highlights the potential variability in disease severity, even among patients with confirmed genetic mutations. Given the underlying genetic basis of SIOD, glucocorticoids were not administered. The patient received anti-proteinuric therapy; however, kidney function progressively declined, leading to ESKD and the initiation of hemodialysis. Moreover, this represents the first genetically confirmed and clinically documented case of SIOD reported in Qatar.

## CONSENT FOR PUBLICATION

Informed consent was obtained from the patient.

## DATA AVAILABILITY STATEMENT

The data supporting the findings of this study are available from the corresponding author upon reasonable request.

## AUTHORS’ CONTRIBUTION

ME: manuscript writing and literature review. AN & EA: manuscript review and patient follow-up. HA: manuscript supervision.

## ACKNOWLEDGMENTS

We sincerely thank the staff at Hamad Medical Corporation for their dedicated efforts.

## COMPETING INTERESTS

The authors have no conflicts of interest to declare.

## Figures and Tables

**Table 1.** Comparison of the present case with the previously published juvenile variants of Schimke immunoosseous dysplasia (SIOD) cases.

Cases	Age at presentation	Gender	Country	History of severe infection	Bone X-ray	Proteinuria	Renal biopsy	Initial IS use	Time to ESKD and modality of dialysis
Hashimoto et al.^17^	16 years	Female	Japan	Absent	Spondyloepiphyseal dysplasia	Nephrotic-range	FSGS	Yes	NA PD
Bakr et al.^18^	14.5 years	Male	Egypt	Present	Flattening of the lumbar vertebrae	Less than 1 g/day	FSGS	Yes	Expired before reaching ESKD
Lama et al.^20^	12.5 years	Female	Italy	Absent	Deformation of the femur’s head, dysplasia of the femoral epiphyses	Less than 1 g/day	Not done (FSGS was reported in two of her brothers)	Yes	Did not reach ESKD till publication
Present case	15 years	Male	Qatar	Absent	Flattened vertebral bodies	Nephrotic-range	FSGS	No	1.5 years HD

ESRD: End-stage kidney disease; FSGS: Focal segmental glomerulosclerosis; HD: Hemodialysis; IS: Immunosuppression; NA: Not applicable; PD: Peritoneal dialysis.
